# Update on the Role of Autologous Hematopoietic Stem Cell Transplantation in Follicular Lymphoma

**DOI:** 10.4084/MJHID.2012.074

**Published:** 2012-11-07

**Authors:** Mónica Cabrero, Alba Redondo, Alejandro Martin, Dolores Caballero

**Affiliations:** Servicio de Hematología: Hospital Universitario e Instituto Biosanitario (IBSAL) de Salamana . Paseo de San Vicente 37007 Salamanca, Spain

## Abstract

Follicular lymphoma (FL) remains incurable despite advances in new strategies of treatment, including monoclonal antibodies (MoAb). Except for early stages, FL is characterized by responses to treatments and systematic relapses. The main objective in this disease is to achieve a better progression free survival (PFS) and to increase overall survival (OS), mainly in young patients. In order to improve the results of conventional chemotherapy, autologous stem cell transplant (ASCT) is a feasible treatment in these patients. In this moment, ASCT is not recommended as first line treatment, except for transformed FL, but is a good strategy as salvage therapy with an improved PFS and OS. New drugs have been introduced to enhance responses of ASCT, but nowadays they are not part of conventional conditioning regimen.

## Indroduction

Follicular lymphoma (FL) is the most common low-grade lymphoma, and the second in frequency of the totality of non-Hodgkin lymphomas (NHL). FL accounts for 25% of newly diagnosed lymphomas. The natural history of this disease is characterized by responses to treatments and systematic relapses.^1^

Introduction of new drugs, mainly Rituximab and other monoclonal antibodies have improved progression free survival (PFS) and overall survival (OS), but nowadays, FL remains incurable.

Intensified regimens including autologous stem cell transplant (ASCT) are feasible strategies in young patients mainly as salvage therapy.

Conventional treatments include radiotherapy for early stages and immune chemotherapy for advances stages. More than 80% of patients have advanced disease at diagnosis, but only those with related symptoms of lymphoma must be treated. In the Rituximab era, an induction regimen including this drug is the choice of treatment. Moreover, recently, the addiction of Rituximab maintenance has improved PFS in both untreated and relapse/refractory FL patients who did not receive prior Rituximab (PRIMA and EORTC studies).^2^,^3^,^4^

## Autologous Stem Cell Transplantation in Follicular Lymphoma

FIRST LINE THERAPY: Prior to the widespread use of Rituximab in first line treatment, high dose chemotherapy followed by ASCT has been tested in young patients. There are 5 randomized clinical trials^4^–^8^ that have shown some improvement in PFS but not in OS. Among these trials there are 2 that include patients who received Rituximab during induction therapy.^7^,^8^ Results in terms of PFS and OS are similar independently of prior exposition to anti-CD20, with similar data in all the published trials. (Table 1).

Before deciding to perform an ASCT as first line therapy, the potential risk of second malignancies like myelodysplastic syndromes, acute myeloid leukemias or solid neoplasm should be considered.^9^

Finally, two meta-analysis (10,11) have evaluated the role of ASCT as first line treatment in FL, and no benefit in OS compared with conventional therapy despite the prolonged PFS observed has been demonstrated.

Based on these results, we can conclude that, despite the benefit on PFS, ASCT is not recommended as first line treatment for FL because there is no evidence of improvement in OS, and a higher risk of second malignancies has been reported.

## Relapse FL

There are many options for relapsed FL patients; we can consider Rituximab alone, combination regimen of chemotherapy and rituximab or radioimmunotherapy. Autologous stem cell transplant has an important role in young relapsed patients.

Prior to the Rituximab era, a phase III randomized clinical trial (The CUP trial)^12^ compared conventional chemotherapy to ASCT in 89 patients with relapsed or progressive FL, and showed a benefit on PFS and OS for patients who received an ASCT. Two years PFS was 26% versus 55%, and 4 years OS was 46% versus 71% for chemotherapy and ASCT respectively.

More recently, several retrospective phase II studies (have shown) showed a longer PFS and OS for ASCT versus chemotherapy regimens.^13^–^18^ Sebban et al. published data of a retrospective analysis of 254 patients previously included in 2 prospective randomized trials from GELA/GELF group, who relapsed after first line therapy. Comparing the treatment at relapse, those patients who have received salvage therapy including Rituximab followed by an ASCT exhibit an enhanced PFS and OS comparing to those receiving only chemotherapy. (Five year overall survival after relapse 70% (95% CI, 59% to 79%) vs. 42% (33% to 50%) and 5-year event free survival after relapse 51% (40% to 61%) vs. 24% (17% to 31%), *p < .0001* in both cases). Although a benefit from ASCT was observed in all patients who received Rituximab as part of salvage therapy, these results have not been confirmed in another patient cohort, so it is not clear yet if the benefit of ASCT remains after including Rituximab as part of the rescue therapy.

The recent study, by Le Gouill et al, includes 175 patients in first relapse FL, previously treated according to a randomized phase III trial (FL2000). This study reports a significant better 3 years OS in ASCT (92%, CI 78–97%) as compared with conventional chemotherapy treatment (63%, CI 51–72%); otherwise, it also showed the benefit of Rituximab treatment in relapsed FL patients both Rituximab naive and prior treated.^19^

In conclusion, according to the available data, ASCT is recommended in first relapse FL, based mainly in pre-Rituximab studies.

## New Drugs in ASCT

Despite that ASCT seems to be the best option for relapse FL, the primary cause of failure of this approach is the disease recurrence. In order to achieve durable remissions and decrease relapse after ASCT with limited toxicity, new drugs have been included as part of conditioning regimen.

## Radioinmunotherapy (RIT)

We know that lymphomas are sensitive to irradiation, so radiotherapy has been used as conditioning regimen in ASCT and more recently the association of radioisotopes with tumour-associated antigens such as CD20 has allowed exploring a new way to improve ASCT’s results by the intensification of tumour irradiation without increasing organ toxicity. Yttrium (90) ibritumomab tiuxetan (Zevalin) and I-131 tositumomab have been tested, and the results of many phase I and II trials suggest that this can be a safety and effective strategy combined with high-dose chemotherapy and further stem cell transplantation.^20^

Nademadee A. et al published in 2005 the results of a phase I/II study combining high dose Y-90 ibritumomab tiuxetan with high dose etoposide (40–60 mg/kg) and cyclophosphamide (100 mg/kg) that included 12 patients with relapse FL.^21^ At a median follow up of 22 months, the 2-year estimated overall survival (OS) was 92% and the disease-free survival was 78%.

More recently, Decaudin D. et al have reported the results of a GELA phase II study to evaluate the safety and efficacy of a conventional dose of (90)Y ibritumomab tiuxetan combined with carmustine, etoposide, cytarabine and melphalan (BEAM) regimen before autologous stem cell transplantation (ASCT) in B-NHL.^22^ Sixty-nine patients with diagnosis of FL were included in the trial. Before ASCT, 77% and 23% of patients were in CR and PR respectively. At day +100, 88% of patients were in CR/CRu, and at a median follow up of 28 months, 2-year event-free survival and OS were 63% and 97%, respectively.

In 2007, Gopal et al. published data from 24 patients older than 60 years who received a myeloablative dose of I-131 tositumomab (monitored by dosimetry) followed by an ASCT.^23^ Having been submitted to a median of four prior regimens, these patients were not considered suitable for conventional conditioning regimen because of its potential toxicity. With a median follow-up of 2.9 years, the estimated 3-year OS and PFS rates were 59% and 51%, respectively.

Although phase III trials comparing radioimmunotherapy with conventional conditioning regimens are not available in this moment, Gopal et al. compared data from FL patients included in phase I/II trials of I-131-tositumomab with 98 historical controls treated with conventional high- dose therapy followed by infusion of autologous stem cell.^24^ Historical controls received total body irradiation plus chemotherapy or chemotherapy alone. After a multivariate analysis, patients who receive radioimmunotherapy had a significant reduction in the risk of the disease progression or death (HR=0.5, p =0.03) compared to the control group. The estimated OS was 67% vs. 53%, and estimated PFS was 48% vs. 29%, for patients receiving radioimmunotherapy and conventional ASCT respectively.

Based on these data we can conclude that RIT is a promising option to improve results of ASCT in FL, but randomized trials are needed to establish the role of these new drugs in salvage therapy.

## Transformed FL

Histologic transformation of follicular lymphoma into an aggressive lymphoma, like diffuse large B-cell lymphoma, implies a worsening of prognosis, including worse response to conventional treatment. Approximately 3% of patients with follicular lymphoma develop transformation annually, clinically characterised by a rapid lymph node growth with increased levels of lactate dehydrogenase or an intensive uptake in positron emission tomography.

Most studies published about ASCT in transformed FL are based on pre-rituximab data, and they show that we can obtain sustained complete remission with this strategy. In the more recent study published in 2011, a prospective phase II by the Norwegian group(25 including patients who did not receive Rituximab in first line treatment, 60% of patients obtained CR after ASCT. With a median follow-up of 75 months, PFS and OS were 26 and 47 months respectively, compared with a median OS of 10 months for patients not eligible for ASCT.

The impact of rituximab has not been established yet, but ASCT seems to remain an effective option for transformed FL.^26^

## Conclusions

Recently, an evidence-based review has been published by a panel of follicular lymphoma experts with the following recommendations:^27^

- ASCT is recommended as salvage therapy based on pre-rituximab data, with a significant improvement in overall survival (OS) and progression-free (PFS) survival. After Rituximab containing salvage therapy, there is no sufficient evidence-based data to recommend ASCT, so more studies are needed to establish the actual role of ASCT in FL.
- ASCT is not recommended as first-line treatment. Despite the prolongation in PFS there is no improvement in OS.
- ASCT is recommended for transformed follicular lymphoma patients.

## Figures and Tables

**Table 1:** Autologous stem cell transplant in FL as first line treatment

	PROTOCOLS		RESULTS	
Lenz et al. 2004 GLSG	Induction: CHOP or MCP ASCT vs Maintenance: IFN ASCT conditioning regimen: TBI+Cy		5 years PFS: ASCT 64.7% Not ASCT 33.3% *p<0.001*	No impact on OS
Deconick et al. 2005 Gyan et al. 2009 GOELAMS	Induction: VCAP/DHAP ASCT vs Maintenance: IFN ASCT conditioning regimen: TBI+Cy	ORR ASCT 81% Not ASCT 69%	Median PFS: Not reached 45 months *p<0.001*	No impact on OS
Sebban et al. 2006 GELA	Induction: CHOP ASCT vs Maintenance: IFN ASCT conditioning regimen: TBI+Cy+VP16	ORR ASCT 79% Not ASCT 78%	No impact on PFS	No impact on OS
Ladetto et al. 2008 GITMO	R-CHOP vs R-HDS + ASCT	CR R-CHOP 62% ASCT 85%	4 years PFS: ASCT 61% Not ASCT 28% *p<0.001*	No impact on OS
